# Supplementary material for: Differences in the Prevalence and Profile of DSM-IV and DSM-5 Alcohol Use Disorders—Results from the Singapore Mental Health Study 2016
Source: Int J Environ Res Public Health. 2022 Dec 24;20(1):285. doi: 10.3390/ijerph20010285 (PMC9819399; doi:10.3390/ijerph20010285)
Supplement: Supplementary file 1 [file ijerph-20-00285-s001.zip › ijerph-1996122-supplementary.pdf]

International Journal of
Environmental Research and Public Health

Supplementary Table S1. Relationship between lifetime DSM-IV and DSM-5 alcohol use disorder and chronic conditions.

	DSM-IV				DSM-5			
	**%**	**OR ***	**95% CI**	***p* Value**	**%**	**OR**	**95% CI**	***p* Value**
**Lifetime chronic conditions**	52.5	1.3	0.9–1.9	0.227	61.4	1.9	1.2–3.3	**0.013**
Hypertension	15.8	1.0	0.6–1.8	0.959	20.1	1.7	0.8–3.6	0.195
Hyperlipidaemia	12.6	0.8	0.4–1.4	0.439	16.4	1.4	0.7–3.0	0.385
Diabetes	5.5	0.7	0.3–1.5	0.333	6.6	1.2	0.4–3.2	0.774
Asthma	15.4	0.9	0.6–1.6	0.803	13.0	0.8	0.4–1.6	0.536
Chronic pain	21.7	1.6	1.03–2.5	**0.035**	34.0	2.9	1.7–4.9	**<0.001**
Cardiovascular diseases	7.2	2.6	1.3–5.5	**0.009**	12.4	5.3	2.2–12.9	**<0.001**
Ulcer	3.2	1.5	0.6–4.0	0.386	1.0	0.5	0.1–1.5	0.202
Thyroid	1.0	0.2	0.1–0.8	**0.016**	1.6	1.1	0.2–6.1	0.920

* Odds Ratio was generated from multiple logistic regression after controlling for significant socio-demographic factors.

Supplementary Table S2. Relationship between DSM-IV and DSM-5 alcohol use disorder and Health-related Quality of Life scores.

	DSM-IV AUD				DSM-5 AUD			
	**Mean (SD)**	**Coef. ***	**95% CI**	***p***	**Mean (SD)**	**Coef. ***	**95% CI**	***p***
SF-12 subdomain Scores								
Physical component summary (PCS)	43.0 (5.2)	1.2	0.3–2.2	**0.010**	43.9 (4.6)	2.1	0.9–3.2	**<0.001**
Mental component summary (MCS)	55.9 (9.4)	−2.8	−4.4–−1.1	**0.001**	55.7 (9.5)	−2.5	-4.8–−0.3	**0.025**

* Coefficient was generated from multiple linear regression after controlling for significant socio-demographic factors. For DSM-IV, the regression model was controlled for age, gender, ethnicity, education, employment and income while for DSM-5 the model was controlled for age, gender, marital and education status.
